# Taxonomy and Phylogeny of Three New Euplotid Ciliates (Ciliophora, Euplotidae) Isolated from High Latitudes

**DOI:** 10.3390/microorganisms14030563

**Published:** 2026-03-01

**Authors:** Huan Li, Mikhail Tribun, Yuxuan Wang, Yunhan Wang, Sitong Li, Chunyu Lian, Xuming Pan

**Affiliations:** 1 Laboratory of Protozoology, Harbin Normal University, Harbin 150025, China; 2 Zoological Institute of the Russian Academy of Sciences, 199034 St. Petersburg, Russia

**Keywords:** biodiversity, molecular, morphology, SSU rDNA

## Abstract

The biodiversity of euplotid ciliates in high-latitude regions has long been underestimated due to a historical research focus on mid- to low-latitude areas. This study describes three new species collected from high latitudes, *Euplotes aspergilliformis* sp. nov., *E. borealis* sp. nov., and *E. verebkovi* sp. nov. *Euplotes aspergilliformis* sp. nov. is characterized by medium body size (60–85 × 40–60 μm). It possesses nine frontoventral cirri, two marginal cirri and two caudal cirri with forked distal ends. It also has eight dorsal kinetids (with 13–17 dikinetids in the mid-dorsal kinety) and a double-*eurystomus* type silverline system. *Euplotes borealis* sp. nov. is a medium-sized (45–55 × 25–35 μm) freshwater ciliate. It can be recognized by nine frontoventral cirri, one marginal and two caudal cirri. It has nine dorsal kineties with about nine basal bodies in the mid-dorsal kinety, and a double-*eurystomus* type silverline system. *Euplotes verebkovi* sp. nov. features an elongated oval body (40–54 × 22–31 μm). It has ten frontoventral, five transverse, one marginal, and two caudal cirri. It possesses seven dorsal kineties with eight or nine bristles in the mid-dorsal row, seven conspicuous dorsal ridges, and a double-*eurystomus* type dorsal silverline system. Phylogenetic analyses based on molecular data revealed the systematic positions of the three new taxa and confirmed the validity of the three organisms as distinct species.

## 1. Background

Euplotid ciliates represent one of the most species-rich groups within the phylum Ciliophora, having garnered considerable attention from protozoologists over the past few decades [1,2,3,4,5,6]. The most speciose genus within this group, *Euplotes* Ehrenberg, 1830, represents one of the most complex and diverse taxa, with a cosmopolitan distribution across freshwater, marine, and terrestrial biotopes [7,8,9,10].

To date, over 160 nominal species have been recognized in *Euplotes* [11,12,13,14,15,16]. Traditionally, species identification in this genus relied primarily on morphological characters of living specimens. With the adoption of integrative taxonomic approaches, additional species-specific characteristics (e.g., ciliary pattern, silverline system) have been incorporated into the identification process [17,18,19]. In recent years, molecular data have also become widely utilized to resolve taxonomic ambiguities and delimit new species [20,21,22,23,24,25,26,27].

Despite this long history of morphological and taxonomic research on euplotids, investigations have predominantly concentrated on mid- to low-latitude regions. In contrast, high-latitude areas characterized by extreme environmental conditions, including prolonged ice cover, low temperatures, and pronounced seasonal fluctuations in light availability, have remained largely unexplored. The extreme selective pressures of high-latitude regions shape unique microbial communities in which *Euplotes* plays a crucial ecological role, and the recent discovery of considerable species diversity there underscores the critical need for more comprehensive surveys [28,29,30,31]. Currently, high-latitude regions remain critically undersampled for ciliate biodiversity, leaving a substantial gap in our understanding of the biogeography and evolutionary adaptations of the genus *Euplotes*. In the present study, we take an initial step toward addressing this gap by describing three new *Euplotes* species isolated from high-latitude areas and elucidating their phylogenetic positions.

## 2. Materials and Methods

### 2.1. Sampling and Morphological Methods

**Figure 1:**
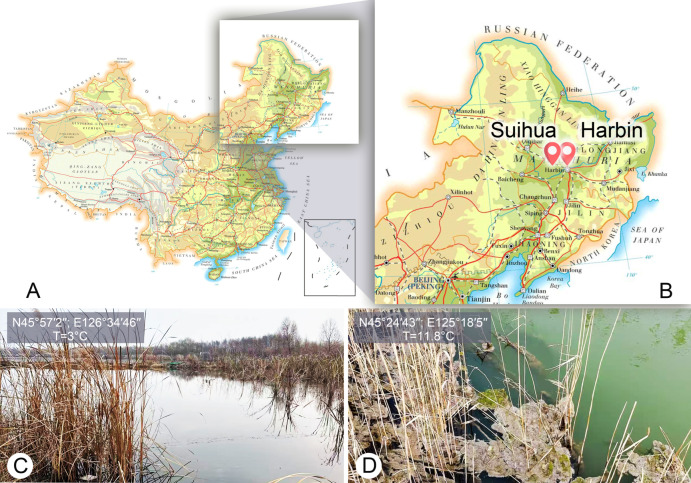
Locations of the sample sites (**A**–**D**). (**A**,**B**) Maps of sampling sites. (**C**) Showing the sampling site and the corresponding ambient environment at Hulan River, Harbin, Heilongjiang Province, China. (**D**) Showing the sampling site and the corresponding ambient environment at a small pond near Anda People’s Court, Suihua, Heilongjiang Province, China.

*Euplotes aspergilliformis* sp. nov. was collected on 22 November 2024 from the Hulan River (45°57′2″ N, 126°34′46″ E), Harbin, Heilongjiang Province, China, where the water temperature was 3 °C (Figure 1A–C). *Euplotes borealis* sp. nov. was found on 7 April 2025 from a small pond near Anda People’s Court (45°24′43″ N, 125°18′5″ E), Suihua, Heilongjiang Province, China, where the water temperature was 11.8 °C (Figure 1A,B,D). *Euplotes verebkovi* sp. nov. was isolated from the private farm “Verebkovo” (57°44′40.8″ N, 27°34′08.1″ E) in the Pskov region of Russia, which specializes in rainbow trout breeding.

Although attempts to establish clonal cultures were unsuccessful, no co-occurring morphologically similar *Euplotes* species were detected in the cultures. Moreover, each protargol-stained preparation, comprising approximately 30–50 individuals, displayed consistent morphological features, providing strong evidence that each isolate represents a single species. Initial cultures were established and maintained at room temperature (about 25 °C) in Petri dishes containing sterile distilled water with rice grains added to enrich the growth of bacteria as a food source for the ciliates. Living cells were observed in vivo using bright field and Nomarski differential interference contrast microscopy at magnifications between 100× and 1000× (Zeiss Axio Imager A2, Gottingen, Germany). The infraciliature and nuclear apparatus were revealed using Feulgen staining and protargol staining [32,33], while dry silver nitrate staining [34] was used to reveal the silverline systems. Drawings of stained specimens were made with the help of photomicrographs. Terminology is mainly according to Curds (1975) [11].

### 2.2. DNA Extraction, PCR Amplification, and Gene Sequencing

Living cells were isolated from the raw culture and repeatedly washed with sterile distilled water to remove potential contamination. The cells were then transferred to a 1.5 mL microfuge tube with a minimum volume of water. Genomic DNA was extracted from one to five cells using the DNeasy Blood and Tissue Kit (Qiagen, Hilden, Germany) following the manufacturer’s instructions. The SSU rRNA gene was amplified with the primers Euk A: 5′-AAC CTG GTT GAT CCT GCC AGT-3′, Euk B: 5′-TGA TCC TTC TGC AGG TTC ACC TAC-3′, F9: 5′-CTGGTTGATCCTGCCAG-3′, and R1513 Hypo: 5′-TGATCCTTCYGCAGGTTC-3′ [35,36]. The PCR conditions for *Euplotes aspergilliformis* and *E. borealis* were as follows: denaturation at 98 °C for 30 s, followed by 18 cycles of amplification (98 °C, 10 s, 69–52 °C touch down, 30 s; 72 °C, 1 min); another 18 cycles amplification (98 °C, 10 s, 51 °C, 30 s; 72 °C, 1 min); and a final extension step at 72 °C for 5 min. For *E. verebkovi*, DNA amplification was performed in a T100 Thermal Cycler (BioRad, Hercules, CA, USA) with the following program: 3 min at 95 °C; 39 cycles of 30 s at 95 °C, 1 min at 55 °C, and 2 min at 72 °C; and a final extension at 72 °C for 6 min. To improve sequence quality, subsequent processing differed between the studied species. For *E. aspergilliformis* and *E. borealis*, the PCR products were either directly sequenced or purified by TIANgel Midi Purification Kit (TIANGEN BIOTECH, Beijing, China), cloned using PMD 18-T vector cloning kit (Takara Biomedicals, Shiga, Japan), and a randomly selected clone was sequenced bidirectionally in Shanghai Sangon Biotechnology Company (Shanghai, China). For *E. verebkovi*, the amplicons were cloned using the InsTAclone PCR Cloning Kit (Fermentas, Carlsbad, CA, USA) according to the manufacturer’s protocol. Colonies were tested by PCR amplification using vector-specific M13 primers. PCR products were sequenced using Sanger sequencing by ABI310 (ABI Prism, Foster City, CA, USA). All sequences were generated using bidirectional sequencing. The quality of the obtained sequences can be observed through the corresponding diagram. Individual sequencing reactions were manually assembled into contigs, with careful reference to the diagram to ensure accuracy.

### 2.3. Phylogenetic Analyses

The newly obtained SSU rDNA gene sequence of three *Euplotes* species and those of 83 other euplotids downloaded from GenBank database were used for phylogeny analyses. Six discocephalids were used as outgroup taxa. Sequences were aligned using MAFFT version 7 server (https://mafft.cbrc.jp/alignment/server/ (accessed on 26 January 2026)). After alignment, the primer binding sites were trimmed manually by Bioedit v.7.2.5 [37], resulting in a final alignment including 2172 sites.

Maximum likelihood (ML) analysis was performed using RAxML-HPC2 on ACCESS v8.2.12 on the online server CIPRES Science Gateway (http://www.phylo.org/portal2/login!input.action (accessed on 26 January 2026)) with the GTRGAMMA model (Miller et al., 2012; Stamatakis, 2014) [38,39]. The reliability of internal branches was assessed using a nonparametric bootstrap method with 1000 replicates. Bayesian inference (BI) analyses were carried out using MrBayes on Access v3.2.x [40] on the CIPRES Science Gateway with GTR + I + G model selected by Akaike Information Criterion (AIC) in MrModeltest v2.2 [41]. Markov chain Monte Carlo (MCMC) simulations were run for 4,000,000 generations, with sampling every 100 generations; the first 10,000 trees were discarded as a burn-in. MEGA 11 v11.0.13 was used to visualize the tree topologies [42].

For interpretation of bootstrap values, we followed the protocol described by Vd’ačný and Rajter [43], that is, values ≥ 95 are considered to be high, 71 to 94 are moderate, 50 to 70 are low, and <50 have no support [44]. Bayesian posterior probabilities < 0.95 are considered as low and values ≥ 0.95 as high [45].

ZooBank Registration of present work: urn:lsid:zoobank.org:pub:C7711067-3EE8-40FF-B748-715C1AF1A025.

## 3. Results

### 3.1. Euplotes aspergilliformis sp. nov. (Figure 2A–M, Table 1)

**Diagnosis:** Freshwater species with a size of 60–85 × 40–60 μm in vivo; outline generally oval-shaped, buccal field about 70% of body length with approximately 34 adoral membranelles; nine frontoventral cirri; two marginal and two caudal cirri; five transverse cirri; consistently eight dorsal kineties with about 15 dikinetids in mid-dorsal kinety; macronucleus C-shaped; double-*eurystomus* type silverline system.

**Locality:** Water sample from the Hulan River (45°57′2″ N, 126°34′46″ E), Harbin, China, where the temperature was about 3 °C.

**Figure 2:**
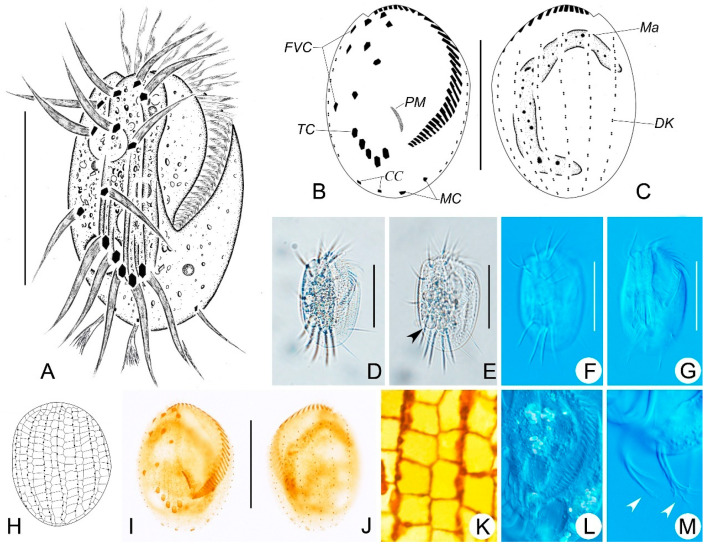
Morphology and infraciliature of *Euplotes aspergilliformis* sp. nov. in vivo (**A**,**D**–**G**,**L**,**M**), after protargol staining (**B**,**C**,**I**,**J**) and dry silver nitrate (**H**,**K**) staining. (**A**,**D**) Ventral views of the representative individual. (**B**,**C**,**I**,**J**) Ventral (**B**,**I**) and dorsal (**C**,**J**) views of the holotype specimen, showing the infraciliature and nuclear apparatus. (**E**,**F**) Ventral views of different individuals; arrowheads indicate contractile vacuole. (**G**) Showing the ridges on ventral sides. (**H**,**K**) Silverline system on dorsal side. (**L**) Showing the endoplasm of a cell. (**M**) Posterior part of an individual; arrowheads show the caudal cirri. Scale bars: 50 μm.

**Materials:** The holotype slide (registration number: LH2024112203-1) with protargol-stained holotype specimens (circled with black ink on the back of slide; Figure 2B,C,I,J), three paratype slides with protargol-stained specimens (Nos. LH2024112203-2, LH2024112203-5, LH2024112203-6), and one paratype slide with dry silver-nitrate-stained specimens (No. LH2024112203-11) are deposited in the Laboratory of Protozoology, Harbin Normal University.

**Etymology:** The species-group name *aspergilliformis* (meaning brush-shaped) refers to the brush-like caudal cirri of the living cells.

ZooBank registration of *E. aspergilliformis*: urn: lsid: zoobank. org: act: urn:lsid:zoobank.org:act:DC2FC703-8892-40F9-9BF8-5DC6761607A7.

**Table 1:** Morphometric data of *Euplotes aspergilliformis* sp. nov. (asp), *Euplotes borealis* sp. nov. (bor) and *Euplotes verebkovi* sp. nov. (ver) after protargol or silver nitrate staining.

Character	Species	Min	Max	Mean	Med	SD	CV	n
Body length in μm	*asp*	63	81	69.7	68	5.9	8.5	19
	*bor*	39	55	46.0	45	4.6	10.0	25
	*ver*	40	54	47.6	47.1	3.7	7.9	20
Body width in μm	*asp*	42	59	50.0	50	5.1	10.2	19
	*bor*	24	37	29.8	29	3.6	12.0	25
	*ver*	22	31	27.2	26.8	2.8	10.4	20
Length of AZM	*asp*	44	52	47.2	47	2.0	4.2	19
	*bor*	26	40	31.2	31	2.9	9.3	25
	*ver*	25	36	31.2	31.6	3.1	9.9	20
No. of AZM	*asp*	32	37	33.8	34	1.2	3.6	19
	*bor*	25	33	28.8	29	2.0	6.9	25
	*ver*	27	34	29.8	29	1.9	6.6	13
No. of FVC	*asp*	9	9	9.0	9	0	0	19
	*bor*	9	9	9.0	9	0	0	25
	*ver*	10	10	10.0	10.0	0	0	14
No. of TC	*asp*	5	5	5.0	5	0	0	19
	*bor*	5	5	5.0	5	0	0	25
	*ver*	5	5	5.0	5	0	0	15
No. of MC	*asp*	2	2	2.0	2	0	0	19
	*bor*	1	1	1.0	1	0	0	25
	*ver*	1	1	1.0	1.0	0	0	14
No. of CC	*asp*	2	2	2.0	2	0	0	19
	*bor*	2	2	2.0	2	0	0	25
	*ver*	2	2	2.0	2.0	0	0	14
No. of DK	*asp*	8	8	8.0	8	0	0	19
	*bor*	9	9	9.0	9	0	0	25
	*ver*	7	7	7.0	7.0	0	0	14
No. of dikinetids in mid-DK	*asp*	13	17	14.9	15	1.1	7.7	19
	*bor*	8	10	8.7	9	0	7.0	25
	*ver*	8	9	8.3	8	0.4	5.8	15
No. of dikinetids in left-DK	*asp*	10	15	12.0	12	1.3	10.8	19
	*bor*	4	6	4.8	5	0	12.5	25
	*ver*	6	10	8.2	8	0.9	11.6	15
No. of dikinetids in right-DK	*asp*	10	14	12.3	12	1.1	9.0	19
	*bor*	7	14	10.3	10	1.9	18.1	25
	*ver*	5	8	6.4	6	1.1	17.4	15

AZM, adoral zone of membranelles; CC, caudal cirri; CV, coefficient of variation in %; DK, dorsal kinetids; FVC, frontoventral cirri; left-DK, leftmost dorsal kinetids; Max, maximum; MC, marginal cirri; Mean, arithmetic mean; Med, median; mid-DK, middle dorsal kinetids; Min, minimum; n, number of cells measured; No., number; right-DK, rightmost dorsal kinety; SD, standard deviation; TC, transverse cirri.

**Morphological Description:**

Cells measuring 60–85 × 40–60 μm in vivo. Both left and right margins are convex, anterior and posterior ends are smoothly rounded (Figure 2A,D–G). Three conspicuous ventral ridges extending posteriorly to transverse cirri with some shorter ridges between them (Figure 2A,G). Cytoplasm is colorless, with numerous food vacuoles of varying sizes in the center, making the cell somewhat opaque in contrast to the highly transparent margin (Figure 2D,E,L). The contractile vacuole is about 11 μm in diameter, located posterior to the rightmost transverse cirrus (Figure 2A,E).

The buccal field is approximately 70% of the body length, composed of 32–37 membranelles, with bases up to 19 μm long (Figure 2A,B,I). Invariably, there are nine frontoventral cirri (cilia about 28 μm long); five transverse cirri, cilia all about 33 μm long; two marginal cirri (cilia about 20 μm long); and two caudal cirri (cilia about 16 μm long) with forked distal ends (Figure 2A,B,I,M). There are eight dorsal kineties, with 13–17 dikinetids in the mid-dorsal kinety and about 12 basal bodies in the leftmost dorsal kinety (Figure 2C,J). It has a dorsal silverline system of double-*eurystomus* type (Figure 2H,K).

### 3.2. Euplotes borealis sp. nov. (Figure 3A–M, Table 1)

**Diagnosis:** Small freshwater *Euplotes*, in vivo about 45–55 × 25–35 μm; generally oval-shaped; buccal field about 75% of body length with approximately 29 adoral membranelles; nine frontoventral cirri; one marginal and two caudal cirri; five transverse cirri; consistently nine dorsolateral kineties with about nine dikinetids in mid-dorsal kinety; macronucleus C-shaped; dorsal silverline system double-*eurystomus* type.

**Locality:** Freshwater from a small pond near Anda People’s Court (45°24′43″ N, 125°15′5″ E), Heilongjiang, China, where the temperature was about 11.8 °C.

**Figure 3:**
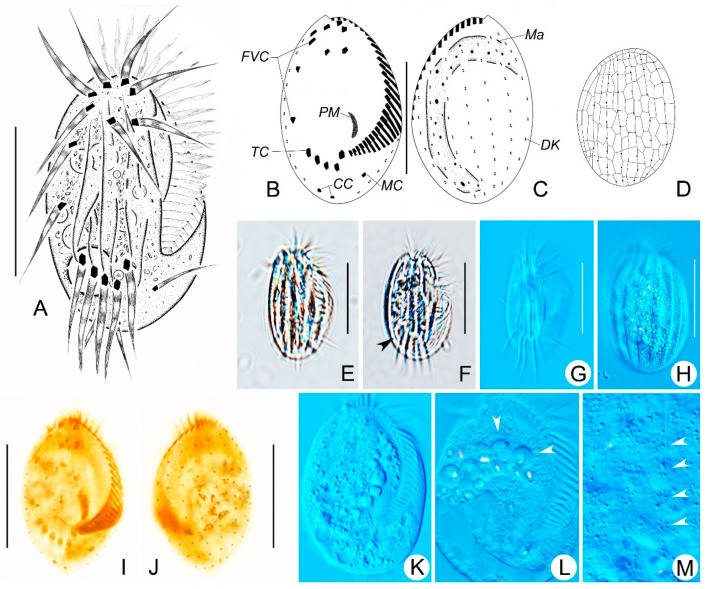
Morphology and infraciliature of *Euplotes borealis* sp. nov. in vivo (**A**,**E**–**H**,**K**–**M**), after protargol staining (**B**,**C**,**I**,**J**) and dry silver nitrate (**D**) staining. (**A**,**E**) Ventral views of the representative individual. (**B**,**C**,**I**,**J**) Ventral (**B**,**I**) and dorsal (**C**,**J**) views of the holotype specimen, showing the infraciliature and unclear apparatus. (**D**) Silverline system on dorsal side. (**F**) Ventral view of a different individual; arrowheads indicate contractile vacuole. (**G**,**H**) Showing the ridges on ventral (**G**) and dorsal (**H**) sides. (**K**,**L**) Showing the endoplasm; arrowheads indicate the food vacuoles. (**M**) Sub-pellicular rosette-like structures around dorsal cilia; arrowheads indicate the basal positions of dorsal cilia. Scale bars: 30 μm.

**Materials:** One slide (No. LH2025040714-1) with protargol-stained holotype specimens (circled with black ink in the back of slide; Figure 3B,C,I,J), four paratype slides (Nos. LH2025040714-2, LH2025040714-3, LH2025040714-4, LH2025040714-5) with protargol-stained holotype specimens, and one paratype slide (No. LH2025040714-17) with the dry silver-nitrate-stained specimens are deposited in the Laboratory of Protozoology, Harbin Normal University.

**Etymology:** The species-group name *borealis* (meaning north) refers to the fact that this species was first described from a northern freshwater habitat (Heilongjiang Province, China).

**ZooBank registration of** ***Euplotes borealis***: urn: lsid: zoobank. org: act: CD9D8E56-EA42-41AF-BD7F-00A6A4159CE1.

**Morphological Description:**

Cells in vivo are about 45–55 × 25–35 μm. Both the left and right margins are convex; the anterior end is narrowly rounded (Figure 3A,E–G). There are six or seven longitudinal ridges on the dorsal side (Figure 3H). About six ellipsoid granules surround each dorsal cilium, forming rosette rows along the dorsal kinety (Figure 3M). The cytoplasm is colorless, with opaque endoplasmic particles in the mid-body region (Figure 3E,F,K,L). The contractile vacuole is 10 μm in diameter, adjacent to the rightmost transverse cirrus, pulsating at intervals of about 30 s (Figure 3A,F).

The adoral zone is approximately 75% of the body length, composed of 25–33 membranelles, with bases up to 12 μm long (Figure 3A,B,I). Invariably, there are nine frontoventral cirri (cilia about 18 μm long); five transverse cirri, cilia all about 19 μm long; one marginal cirrus (cilia about 14 μm long); and two caudal cirri with cilia about 12 μm long (Figure 3A,B,I). There are nine dorsal kineties, with 8–10 dikinetids in the mid-dorsal kinety and about five basal bodies in the leftmost dorsal kinety (Figure 3C,J). It has a double-*eurystomus* type of dorsal silverline system (Figure 3D).

### 3.3. Euplotes verebkovi sp. nov. (Figure 4A–D, Table 1)

**Diagnosis:** Medium-sized freshwater *Euplotes* ciliates, in vivo about 40–54 × 22–31 µm. The cell shape is an elongated oval with rounded ends. The buccal field is about 2/3 of the cell length, with about 30 adoral membranelles. The macronucleus is C-shaped, and the micronucleus is spherical. There are seven conspicuous dorsal ridges and seven dorsal kineties, with eight or nine bristles in the central row. It has a dorsal silverline system of double-*eurystomus* type. There are ten frontoventral cirri, five transverse cirri, two caudal cirri and one marginal cirrus.

**Locality:** Private trout farm “Verebkovo” (57°44′40.8″ N; 27°34′08.1″ E) in the Pskov region, Russia.

**Materials:** Slides with silver nitrate-impregnated cells and Feulgen-stained cells of the *E. verebkovi* sp. nov. are available from the Zoological Institute RAS (Laboratory of Cellular and Molecular Protistology).

**Figure 4:**
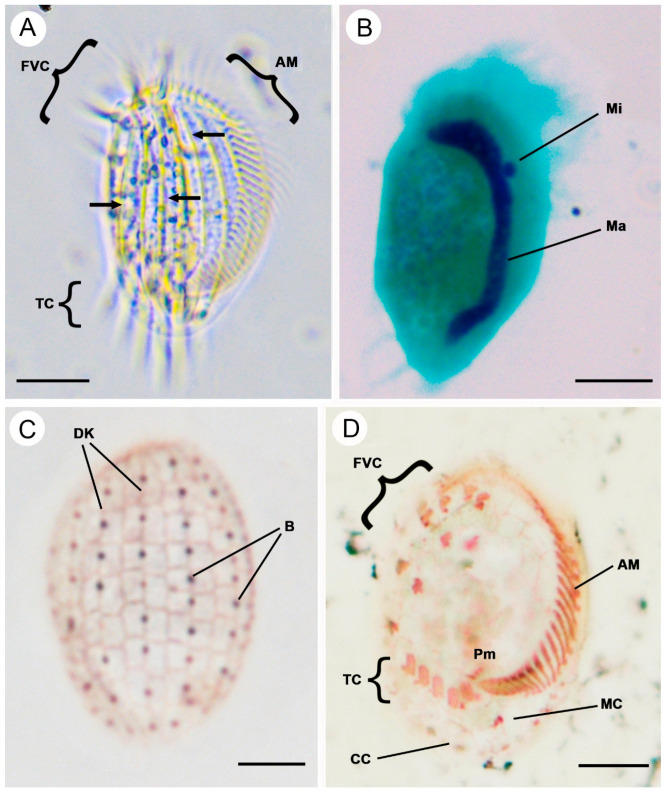
Morphology and infraciliature of *Euplotes verebkovi* sp. nov. in vivo (**A**), after Feulgen (**B**), and silver nitrate impregnation (**C**,**D**) staining. (**A**) Ventral view of the representative individual; arrows show the dorsal ridges. (**B**) Nuclear apparatus stained after Feulgen. (**C**,**D**) Dorsal (**C**) and ventral (**D**) views of representative specimens after silver nitrate impregnation. AM, adoral zone of membranelles; B, bristles; CC, caudal cirri; DK, dorsal kineties; FVC, frontoventral cirri; Ma, macronucleus; MC, marginal cirri; Mi, micronucleus; Pm, paroral membrane; TC, transverse cirri. Scale bars: 10 μm.

**Etymology:** The species name refers to the area where the sample was collected (trout farm “Verebkovo”).

**ZooBank registration of** ***Euplotes verebkovi:*** urn:lsid:zoobank.org:act:7944DF1C-DA95-489A-B0B1-078FC668E277.

**Morphological Description:**

Cells in vivo are about 40.3–54.0 µm in length and about 22.3–31.4 µm in width (average 47.6 ± 3.7 × 27.2 ± 2.8 µm), with a body length/width ratio of about 1.7–1.8. It generally has an elongated oval shape with rounded ends. The dorsal side is decorated with seven ridges (Figure 4A). The cytoplasm is colorless and highly transparent at the marginal area (Figure 4A). The contractile vacuole is not visible. The macronucleus is C-shaped with rounded ends (Figure 4B). The micronucleus is single, spherical (compact type), and usually localized in the cavity of the macronucleus (Figure 4B).

The adoral zone comprises 27–34 membranelles (Figure 4A,D). The ventral cirral pattern is stable, composed of invariably ten frontoventral cirri, five transverse cirri, one marginal cirrus, and two caudal cirri (Figure 4D). The dorsal surface bears seven kineties, with eight or nine bristles in the mid-dorsal row, the leftmost row consists of six to ten bristles and the rightmost row consists of five to eight bristles (Figure 4C). The dorsal argyrome is of the double-*eurystomus* type (Figure 4C).

### 3.4. SSU rDNA Sequences and Phylogenetic Analyses

The small subunit ribosomal RNA (SSU rRNA) gene sequences of *Euplotes verebkovi*, *E. aspergilliformis*, and *E. borealis* have been deposited in GenBank under the accession numbers PQ656668, PZ068345, and PZ068344, respectively. The length and GC contents are as follows: *E. verebkovi* (1996 bp, 43.24%), *E*. *aspergilliformis* (1790 bp, 42.79%), and *E. borealis* (1887 bp, 43.19%).

**Figure 5:**
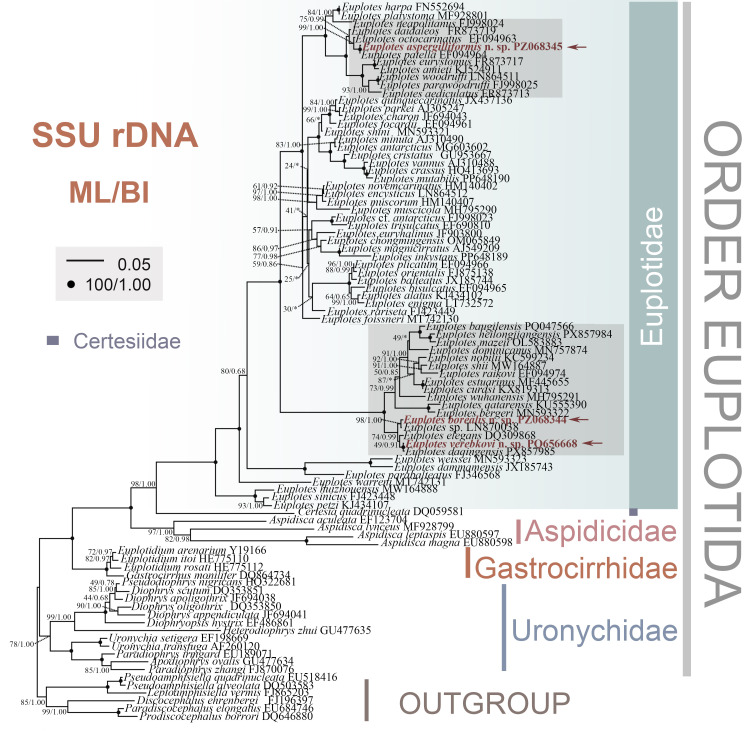
The maximum likelihood (ML) tree inferred from SSU rDNA sequences, showing the phylogenetic positions of *Euplotes aspergilliformis*, *E. borealis* and *E. verebkovi*. Numbers at nodes represent the bootstrap values of the ML analysis and the posterior probability of the Bayesian inference method. Fully supported (100 ML/1.00 BI) branches are marked with solid circles. Asterisk indicates a disagreement between BI tree and the reference ML tree. Scale bar corresponds to 5 substitutions per 100 nucleotide positions. All branches are drawn to scale.

Phylogenetic trees were constructed based on the SSU rRNA gene sequences using both maximum likelihood (ML) and Bayesian inference (BI) methods, with a broad selection of taxa in the order Euplotida. The topologies of ML and BI trees were almost identical; thus, only the ML tree with supported values from both methods is presented here (Figure 5).

Consistent with previous studies, the four families of Euplotida are all monophyletic. It should be noted that the monophyly of Certesiidae could not be assessed, as only one species was included. The monophyly of the family Euplotidae received full support (100 ML/1.00 BI). With full support (100 ML/1.00 BI), the newly obtained *E. aspergilliformis* groups with *E. patella* (EF094964) and clusters tightly with *E. octocarinatus* (EF094963, 99 ML/1.00 BI). The newly sequenced *E. verebkovi* grouped with *E. daqingensis* with full support (100 ML/1.00 BI) and then formed a no-support clade (49 ML/0.91 BI) with *E. elegans* (DQ309868). *Euplotes borealis* (PZ068344) and *Euplotes* sp. (LN870038) are positioned outside this clade with moderate support (74 ML/0.99 BI).

## 4. Discussion

### 4.1. Euplotes aspergilliformis sp. nov.

**Comparison with related congeners.** Considering the double-*eurystomus* type silverline system, nine frontoventral and two marginal cirri, six *Euplotes* species should be compared with *E. aspergilliformis* sp. nov., namely, *E. amieti* Dragesco, 1970, *E. paramieti* Han et al., 2024, *E. woodruffi* Gaw, 1939, *E. eurystomus* (Wrzesniowski, 1870) Kahl, 1932, *E. aediculatus* Pierson, 1943, and *E. finki* Foissner, 1982 (Table 2).

*Euplotes amieti* can be clearly separated from *E. aspergilliformis* sp. nov. by its (1) triangular-shaped adoral zone (vs. nearly C-shaped), (2) 3-shaped macronucleus (vs. inverted C-shaped), (3) larger cell size (130–240 × 70–160 vs. 60–85 × 40–60), (4) higher number of adoral membranelles (52–70 vs. 32–37), (5) more dorsal kineties (12–15 vs. eight), and (6) more basal bodies in the middle kinety (18–32 vs. 13–17) [46,47,48,49,50].

*Euplotes paramieti* differs from *E. aspergilliformis* sp. nov. in (1) the appearance of the adoral zone (proximal ventral membranelles arranged in sigmoidal shape, vs. evenly bending, nearly C-shaped), (2) the shape of the macronucleus (3-shaped vs. inverted C-shaped), (3) cell size (180–220 × 110–155 vs. 60–85 × 40–60), (4) the number of adoral membranelles (63–93 vs. 32–37), (5) the number of dorsal kineties (12–13 vs. eight), and (6) the number of basal bodies in the middle kinety (24–37 vs. 13–17) [15].

*Euplotes eurystomus* can be clearly distinguished from *E. aspergilliformis* sp. nov. by (1) the shape of the adoral zone (conspicuous collar positioned at anterior end vs. evenly bending, nearly C-shaped), (2) the shape of the macronucleus (3-shaped vs. C-shaped), (3) longer cell (88–180 vs. 60–85), (4) higher number of adoral membranelles (44–65 vs. 32–37), and (5) basal bodies in the middle kinety (15–31 vs. 13–17) [11,22,51,52,53,54,55,56,57,58,59].

*Euplotes finki* differs from *E. aspergilliformis* sp. nov. in (1) the biotope (soil vs. freshwater), (2) the arrangement of transverse cirri (a gap between the left two and the remaining three divided them into two groups vs. a continuous group), (3) the shape of caudal cirri (absence vs. presence of a forked distal), (4) the number of adoral membranelles (19–22 vs. 32–37), (5) the number of dorsal kineties (seven vs. eight), and (6) the number of basal bodies in the middle kinety (about 10 vs. 13–17) [60].

*Euplotes woodruffi* differs from *E. aspergilliformis* sp. nov. in (1) the shape of the adoral zone (conspicuous collar positioned at anterior end vs. evenly bending, nearly C-shaped), (2) the shape of the macronucleus (T-shaped vs. C-shaped), (3) larger cell size (83–200 × 58–130 vs. 60–85 × 40–60), (4) greater number of adoral membranelles (40–99 vs. 32–37), (5) higher number of dorsal kineties (8–11, normally nine or ten vs. eight), and (6) greater number of basal bodies in the middle kinety (17–35 vs. 13–17) [22,48,49,56,59,61,62,63,64,65,66].

*Euplotes aediculatus* can be distinguished from *E. aspergilliformis* sp. nov. due to (1) the appearance of the adoral zone (triangular-shaped vs. evenly bending, nearly C-shaped), (2) the shape of the macronucleus (3-shaped vs. C-shaped), (3) larger cell size (105–170 × 60–120 vs. 60–85 × 40–60), (4) a higher number of adoral membranelles (40–70 vs. 32–37), and (5) a higher number of basal bodies in the middle kinety (18–30 vs. 13–17) [22,49,59,62,67,68,69,70,71].

**Table 2:** Comparison of *Euplotes aspergilliformis* sp. nov. with those related congeners having nine frontoventral, two marginal and two caudal cirri.

	Body Size	AZM, n	DK, n	Mid-DK, n	Ma	Isolation Location	Original Description
*E. aspergilliformis*	60–85 × 40–60	32–37	8	13–17	C	FW, China	Present
*E. amieti*	140–240 × 80–160	52–62	14	28 *	3	Cameroon	[46]
	ca. 180 × ?		13			Chad	[47]
	140–240 × ?	52–62	14	26–31	3	Cameroon	[48]
	ca. 169 × ?	53–67	12–15	21–28	3	Rwanda	[49]
	130–200 × 70–100	60–65	12–14	18–26	3	FW, China	[50]
*E. paramieti*	180–220 × 110–155	63–93	12–13	24–37	3	FW, China	[15]
*E. eurystomus*	105–160 × ?	56 *	8	32 *	3	FW	[57]
	121.5–135 × 73.5–85.5	48–52	8–9	28–31	C	FW, USA	[58]
	100–160 × 40–90	50–65	8–12	17–25	3	FW	[11]
	140–180 × 95–135	45–55	9–11	27 *	3	FW, USA	[59]
	88–125 × 55–78	44–53	8–9	15–23	C or 3	FW, China	[52]
	98–133 × 67.2–105	35–40			3	Soil, Paithan	[53]
*E. finki*	60 × 40	19–22	7	10	C or J	Soil, Austria	[60]
*E. woodruffi*	120–165 × 90				T		[62]
	128–150 × 75–95	51–58	9	24–30	T	FW, USA	[59]
	83–140 × 58–90	53–99	8–9	30 *	T	BW, Africa	[48]
	130–160 × 90–110	57–64	10	23–28	T	FW, China	[65]
	110–200 × 70–130	76–85	9	28–35	S	BW, USA	[65]
	130–153 × 81–95	ca. 60	10–11		T	FW, China	[64]
	90–140 × 60–90	56–67	9	17–24	T *	Marine, China	[66]
	110–160 × 60–80	59–70	9–10	19–24	T or Y	FW, China	[61]
	100–1132 × 68–82	40–56	9–10	20–21	T	FW, India	[22]
*E. aediculatus*	130.7–133.3 × 82.2–85.2				C	BW or FW, USA	[62]
		40	8		C	BW or FW	[67]
	105–122 × 65–77	43–51	8–9	18–26	C	FW, USA	[59]
	105–165 × 60–110	40–60	8–9	24 *	3		[69]
	120–130 × 80–85	40–50	8		C	FW, China	[68]
	150–170 × 100–120	47–55	8	21–26	C	FW, China	[70]
	105–165 × 60–110	48–50	8	24–30		FW, Slovakia	[71]
	107–119 × 72–82	42–46	8	18–24	3	FW, India	[22]
*E. indica*	49–52 × 40–46	20–25	7	11–16	C	FW, India	[22]

Abbreviations: 3, 3-shaped; AZM, adoral membranelles; BW, brackish water; C, C-shaped; DK, dorsal kineties; FW, freshwater; J, J-shaped; Ma, macronucleus; mid-DK, middle dorsal kinety; S, S-shaped; T, T-shaped; Y, Y-shaped. * Counted from published illustrations.

*Euplotes indica* differs from *E. aspergilliformis* sp. nov. in (1) the location of marginal cirri (post-orally located, clearly separated from two caudal cirri vs. more subcaudally located, near the caudal cirri), (2) smaller body size in vivo (49–52 × 40–46 vs. 60–85 × 40–60), (3) lower number of adoral membranelles (20–25 vs. 32–37), and (4) lower number of dorsal kineties (seven vs. eight) [22].

### 4.2. Euplotes borealis sp. nov.

**Comparison with related congeners.** In terms of the double-*eurystomus* type silverline system, nine frontoventral cirri, and one marginal cirrus, seven *Euplotes* species are considered relevant for comparison with *E. borealis* sp. nov., namely *E. mazeii* Lian et al., 2023, *E. dogieli* Agamaliev, 1967, *E. bisulcatus* Kahl, 1932, *E. affinis* (Dujardin, 1841) Perty, 1852, *E. foissneri* Valbonesi et al., 2021, *E. warreni* Valbonesi et al., 2021, and *E. nana* Jones & Owen, 1974 (Table 3).

*E. mazeii* can be clearly distinguished from *E. borealis* sp. nov. by (1) the appearance of the adoral zone (evenly bending, nearly C-shaped vs. bent almost 90° at the posterior end), (2) a slightly lower number of adoral membranelles (18–26 vs. 25–33), and (3) a fewer number of dorsal kineties (seven vs. nine) [16].

*Euplotes dogieli* differs from *E. borealis* sp. nov. in the following respects: (1) body shape (right side rectilinear and left side convex vs. generally oval-shaped with both left and right side convex), (2) location of the marginal circus (more subcaudal, near the caudal cirri vs. post-oral, clearly separated from two caudal cirri), (3) body length (ca. 65 vs. 45–55), (4) number of adoral membranelles (35–38 vs. 25–33), (5) number of dorsal kineties (seven vs. nine) and (6) basal bodies in the middle kinety (ca. 13 vs. 8–10) [72].

*Euplotes bisulcatus* can be clearly distinguished from *E. borealis* sp. nov. by: (1) biotope (marine vs. freshwater), (2) the number of adoral membranelles (ca.17 vs. 25–33), (3) a fewer number of dorsal kineties (eight vs. nine), (4) a lower number of dikinetids in middle dorsal kinety (five to seven vs. 8–10), and (5) a lower number of dikinetids in the left dorsal kinety (ca. two vs. four to six) [73,74].

*Euplotes affinis* was first described by Dujardin from a freshwater population. Subsequently, Kahl reported *E. affinis* forma *tricirratus*, which is characterized by a smaller body size and the presence of only one marginal cirrus. Later, Curds provided a redescription of *E. affinis* based on a British population, which closely resembled the population reported by Kahl and supplemented the illustrative diagrams of the silverline system. Given its possession of nine frontoventral, one marginal cirri, and a double-*eurystomus* type silverline system, *E. affinis* forma *tricirratus* also should be compared with *E. borealis* sp. nov. *Euplotes affinis* forma *tricirratus* can be clearly separated from *E. borealis* sp. nov. by (1) the shape of the macronucleus (3-shaped vs. C-shaped), (2) a lower number of adoral membranelles (18–20 vs. 25–33), and (3) a lower number of dorsal kineties (seven vs. nine) [73,75,76].

*Euplotes foissneri* can be clearly distinguished from *E. borealis* sp. nov. by: (1) the arrangement of caudal cirri (aggregated vs. separated), (2) the biotope (brackish water vs. freshwater), (3) the number of dorsal kineties (eight vs. nine), and (4) dikinetids in the middle dorsal kinety (12–16 vs. 8–10) [19].

*Euplotes warreni* can be separated from *E. borealis* sp. nov. due to its (1) body shape (D-shape vs. ovoid), (2) biotope (marine vs. freshwater), (3) a lower number of adoral membranelles (23–25 vs. 25–33), and (4) a lower number of dorsal kineties (six vs. nine) [19].

*Euplotes nana* Jone & Owen, 1974 can be distinguished from *E. borealis* sp. nov. by: (1) the position of the contractive vacuole (situated medially near the posterior body end vs. located posteriorly near right body margin), (2) the biotope (marine vs. freshwater), (3) the location of the marginal circus (more subcaudally, near the caudal cirri vs. post-orally located, clearly separated from the caudal cirri), (4) a lower number of dorsal kineties (ca. eight vs. nine), and (5) a lower number of dikinetids in the middle dorsal kinety (ca. five vs. 8–10) [77].

**Table 3:** Comparison of *Euplotes borealis* sp. nov. with those related congeners having nine frontoventral, two marginal and one caudal cirri.

	Body Size	AZM, n	DK, n	Mid-DK, n	Ma	Isolation Location	Original
*E. borealis*	35–55 × 20–40	25–33	9	8–10	C	FW, China	Present
*E.* *mazeii*	40–55 × 25–35	18–26	7	6–9	C	FW, China	[16]
*E.* *dogieli*	65 ** × ?	35–38	7	13 *	C *	Caspian Sea	[72]
*E. bisulcatus*	80–90 * × ?				S *	Germany	[73]
	34–45 × 27–31	17 *	8	5–7	C *	Tidal marsh pond, USA	[74]
*E. affinis* forma tricirratus	45 × ?					FW	[73]
*E. affinis*	38 × 26	18–20	7	Max 9	3	FW, UK	[76]
*E. foissneri*	46–58 × 32–40	28–32	8	12–16	hook- to a horseshoe-shaped or 3	BW in Chile	[19]
*E. warreni*	34–45 × 25–30	23–25	6	9–11	hook-shaped or C	Marine, Chile	[19]
*E.* *nana*	25–30 × ?	25 *	8 *	5 *	C	Mobile Bay, USA	[77]

Abbreviations: 3, 3-shaped; AZM, adoral membranelles; BW, brackish water; C, C-shaped; DK, dorsal kineties; FW, freshwater; Ma, macronucleus; mid-DK, middle dorsal kinety; S, S-shaped. * Counted from published illustration. ** According to the description, the body length of *Euplotes bisulcatus* was similar to *E. elegans*; this number is based on the body length of *E. elegans*.

### 4.3. Euplotes verebkovi sp. nov.

**Comparison with related congeners.** In terms of morphological and molecular data, five *Euplotes* species are considered relevant for comparison with *E. verebkovi* sp. nov., namely *E. elegans* Kahl, 1932, *E. nobilii* Valbonesi & Luporini, 1990, *E. qatarensis* Fotedar et al., 2016, *E. curdsi* Syberg-Olsen et al., 2016, and *E. daqingensis* Lian et al., 2026 (Table 4).

*Euplotes elegans* can be separated from *E. verebkovi* by its larger body size (87–118 × 43–59 vs. 40–54 × 22–31), more membranelles in the adoral zone (47–64 vs. 27–34), more dorsolateral kineties (nine or ten vs. seven), more bristles in the central row (15–20 vs. eight or nine), and the type of habitat (marine vs. freshwater). Also, the authors mention that *E. elegans* has nine frontoventral cirri and one reduced cirrus. *Euplotes verebkovi* sp. nov. has ten normal-sized frontoventral cirri [9].

*Euplotes nobilii* differs from *E. verebkovi* sp. nov. in the cell shape (spindle-shaped vs. elongated oval), number of membranelles in the adoral zone (18–22 vs. 27–34), dargyrome type (double-*patella* vs. double-*eurystomus*), number of dorsal ridges (six vs. seven), number of dorsolateral kineties (eight vs. seven), and the type of habitat (marine vs. freshwater) [29].

The newly described species can be distinguished from *Euplotes qatarensis* based on the following differences: number of dorsolateral kineties (seven vs. 10), number of marginal cirri (one vs. two), and habitat type (freshwater vs. marine) [78].

*Euplotes curdsi* can be separated from *E. verebkovi* sp. nov. in the number of dorsal ridges (five or six vs. seven), the number of bristles in the central row (10–12 vs. eight or nine), the number of marginal cirri (two vs. one), and the type of habitat (brackish and marine vs. freshwater) [79].

*Euplotes daqingensis*, discovered by Lian et al., despite its close phylogenetic position to *E. verebkovi* sp. nov., has a number of significant morphological differences. Firstly, the number of frontoventral cirri (nine vs. ten). Secondly, the number of dorsal kineties (eight vs. seven) and the number of dorsal ridges (five vs. seven) [80].

### 4.4. Phylogenetic Analyses

The newly sequenced *Euplotes aspergilliformis* is closely related to *E. patella* and *E. octocarinatus* in our ML and BI trees; this corresponds well with the similar morphological characteristics between these three species. The sequence of *E. aspergilliformis* differs in only two nucleotides from the sequence of *E. patella* (compared with EF094964, isolated from Japan rather than the type locality) [81]. Meanwhile, *E. patella* and *E. aspergilliformis* are very similar in cell size and shape, biotope, number of adoral membranelles, the shape of the adoral zone, and the general infraciliature on the ventral side. The most significant difference between *E. aspergilliformis* and *E. patella* is the number of dorsal kineties (eight in *E. aspergilliformis* vs. nine in *E. patella*) and the type of silverline system (double-*eurystomus* type in *E. aspergilliformis* vs. double-*patella* type in *E. patella*), which can be detected only after protargol impregnation and silver nitrate staining. Furthermore, no morphological information is available regarding the sequence of *E. patella* (EF094964), so misidentification cannot be excluded. In the meantime, *E. aspergilliformis* also differs from *E. octocarinatus* in that the new species has an evenly bending adoral zone (vs. nearly triangular in shape) and a double-*eurystomus* type silverline system (vs. double-*patella* type), and the 19 bp sequence differences between them further support the separation of these two species.

*Euplotes borealis* is closely related to *E. elegans*, *E. daqingensis*, *E. verebkovi*, and *Euplotes* sp. (LN870038) in both trees. Among these species, the newly obtained *E. borealis* is most closely related to *Euplotes* sp. (LN870038), sharing 99.57% sequence similarity with only 8 nucleotide differences. Since neither a detailed description of a living organism nor morphometric data was reported for the sequence of *Euplotes* sp. (LN870038), their conspecificity cannot be excluded. In contrast, the remaining species show distinct morphological differences both in vivo and after protargol impregnation, as well as sequence variations exceeding 23 bp, thereby supporting the identification of the new species.

The new species *Euplotes verebkovi* in the present study shows high sequence similarity to *E. daqingensis*, with a genetic divergence of only four nucleotides. Despite this close genetic relationship, they are considered distinct species due to clear morphological differences [80]. The other molecular closely related species, *Euplotes elegans* (DQ309868), exhibits an 18 bp nucleotide difference and clear morphological distinctions, that is, the presence of nine (vs. ten) frontoventral cirri and only one (vs. two) marginal cirrus. Therefore, the phylogenetic analyses further support the establishment of *Euplotes verebkovi* as a new and distinct species.

Recently, many studies have inferred the evolutionary trajectories among *Euplotes* species using various morphological characteristics and genetic markers [82,83]. However, the key morphological evolutionary lineages remain to be clearly identified. Currently accepted morphological evolutionary clues include the pattern of the silverline system, habitat, and infraciliature. In the present studies, all three species examined are freshwater *Euplotes* possessing double-*eurystomus* silverline type; nevertheless, only *E. borealis* and *E. verebkovi* exhibit a close phylogenetic relationship. This finding seems consistent with the fact that these two species have only one marginal cirrus. However, other species with one marginal cirrus are scattered throughout the phylogenetic tree, indicating that this morphological appearance alone is not evolutionarily informative. Therefore, a single morphological feature is not a reliable indicator for reconstructing the evolutionary pathway of the genus *Euplotes*. Further attempts incorporating additional evidence may be necessary in future research.

## 5. Conclusions

As a result of historical research deficiencies, our understanding of euplotid ciliate diversity in high-latitude regions still lags considerably behind that of mid- to low-latitude areas. Although the scientific significance of these regions has now been increasingly recognized, filling this knowledge void will require sustained and long-term efforts. The present study contributes to this ongoing endeavor by providing evidence for the underestimated species diversity of protists in high-latitude habitats and underscoring the necessity for further exploration in these extreme environments.

## Figures and Tables

**Table 4:** Morphological comparison between *Euplotes verebkovi* sp. nov. and some phylogenetic or morphologically related species.

	Body Size, μm	AZM, n	DK, n	Mid-DK, n	FVC, n	Dorsal Silverline System	Dorsal Ridges, n	Isolation Location	Original Description
*E. verebkovi*	40–54 × 22–31	27–34	7	8–9	10	double-*eurystomus*	7	FW, Russia	Present
*E. elegans*	87–118 × 43–59	47–64	9–10	15–20	9 + 1	double-*eurystomus*	7–8	Marine, Denmark	[9]
*E. nobilii*	29–43 × 18–29	18–22	8	8–9	10	double-*patella*	6	Marine, Ross Sea	[29]
*E. qatarensis*	44–70 × 26–57	32–39	10	9–12	10	double-*eurystomus*	7	Marine, Qatar	[78]
*E. curdsi*	45–65 × ?	25–34	6–7	10–12	10	double-*eurystomus*	5–6	Marine, White and Mediterranean Sea	[79]
*E. daqingensis*	32–45 × 19–29	22–27	8	8–12	9	double-*eurystomus*	5	FW, China	[80]

Abbreviations: AZM, adoral membranelles; DK, dorsal kineties; FVC, frontoventral cirri; FW, freshwater; Mid-DK, middle dorsal kinety.

## Data Availability

The original contributions presented in this study are included in the article. Further inquiries can be directed to the corresponding author(s).

## References

[1] Song W., Wilbert N., Chen Z., Shi X. (2004). Considerations on the systematic position of *Uronychia* and related euplotids based on the data of ontogeny and 18S rRNA gene sequence analyses, with morphogenetic redescription of *Uronychia setigera* Calkins, 1902 (Ciliophora: Euplotida). Acta Protozool..

[2] Shen Z., Yi Z., Warren A. (2011). The morphology, ontogeny, and small subunit rRNA gene sequence analysis of *Diophrys parappendiculata* n. sp. (Protozoa, Ciliophora, Euplotida), a new marine ciliate from coastal waters of southern China. J. Eukaryot. Microbiol..

[3] Shen Z., Huang J., Lin X., Yi Z., Li J., Song W. (2010). Morphological and molecular characterization of *Aspidisca hongkongensis* spec. nov. (Ciliophora, Euplotida) from the South China Sea. Eur. J. Protistol..

[4] Park M., Kim S., Min G. (2010). First record of two *Euplotes ciliates* (Ciliophora: Spirotrichea: Euplotida) from Korea. Korean J. Syst. Zool..

[5] Modeo L., Petroni G., Lobban C.S., Verni F., Vannini C. (2013). Morphological, ultrastructural, and molecular characterization of *Euplotidium rosati* n. sp. (Ciliophora, Euplotida) from Guam. J. Eukaryot. Microbiol..

[6] La Terza A., Papa G., Miceli C., Luporini P. (2001). Divergence between two Antarctic species of the ciliate *Euplotes*, *E. focardii* and *E. nobilii*, in the expression of heat-shock protein 70 genes. Mol. Ecol..

[7] Kwon C., Kang Y., Shin M. (2007). Two newly recorded estuarine ciliates, *Euplotes vannus* and *E. parawoodruffi* (Ciliophora: Spirotrichea: Euplotida) from Korea. Korean J. Syst. Zool..

[8] Kim E., Lee W.J. (2019). Redescriptions of *Euplotes encysticus* and *E. rariseta* (Protist: Ciliophora: Euplotida). J. Species Res..

[9] Schwarz M.V.J., Zuendorf A., Stoeck T. (2007). Morphology, ultrastructure, molecular phylogeny, and autecology of *Euplotes elegans* Kahl, 1932 (Hypotrichida; Euplotidae) isolated from the anoxic Mariager Fjord, Denmark. J. Eukaryot. Microbiol..

[10] Di Giuseppe G., Erra F., Frontini F.P., Dini F., Vallesi A., Luporini P. (2014). Improved description of the bipolar ciliate, *Euplotes petzi*, and definition of its basal position in the *Euplotes* phylogenetic tree. Eur. J. Protistol..

[11] Curds C.R. (1975). A Guide to the species of the genus *Euplotes* (Hypotrichida, Ciliatea). Bull. Br. Mus. (Nat. Hist.) Zool..

[12] Aliev A.R. (1987). Morphology of new and poorly studied species of the genus *Euplotes* (Hypotrichida: Euplotidae) from natural water bodies of Azerbaijan. Zool. Zh..

[13] Song W., Warren A., Hu X. (2009). Free-Living Ciliates in the Bohai and Yellow Seas, China.

[14] Méndez-Sánchez D., Mayén-Estrada R., Hu X. (2020). *Euplotes octocarinatus* Carter, 1972 (Ciliophora, Spirotrichea, Euplotidae): Considerations on its morphology, phylogeny, and biogeography. Eur. J. Protistol..

[15] Han K., Pan H., Jiang J. (2022). Taxonomy and SSU rRNA gene-based phylogeny of two new *Euplotes* species from China: *E. chongmingensis* n. sp. and *E. paramieti* n. sp. (Protista, Ciliophora). BMC Microbiol..

[16] Lian C., Jiang J., Xi M., Dong J., Ma H., Al-Farraj S.A., Stoeck T., Wang C., Shao C. (2023). Report and phylogeny of a novel *Euplotes* species from China: *E. mazeii* n. sp., and review of *E. balteatus* (Alveolata, Ciliophora). J. Ocean Univ. China.

[17] Liu W., Jiang J., Tan Y., Lin X. (2020). Novel contributions to the taxonomy of the ciliates genus *Euplotes* (Ciliophora, Euplotida): Redescription of two poorly known species, with a brief note on the distributions of this genus in coastal waters of southern China. Front. Mar. Sci..

[18] Živaljić S., Scherwass A., Schoenle A., Hohlfeld M., Quintela-Alonso P., Nitsche F., Arndt H. (2020). A barotolerant ciliate isolated from the abyssal deep sea of the North Atlantic: *Euplotes dominicanus* sp. n. (Ciliophora, Euplotia). Eur. J. Protistol..

[19] Valbonesi A., Di Giuseppe G., Vallesi A., Luporini P. (2021). Two new species of *Euplotes* with cirrotype-9, *Euplotes foissneri* sp. nov. and *Euplotes warreni* sp. nov. (Ciliophora, Spirotrichea, Euplotida), from the coasts of Patagonia: Implications from their distant, early and late branching in the *Euplotes* phylogenetic tree. Int. J. Syst. Evol. Microbiol..

[20] Tribun M. (2024). Morphology and systematic position of *Euplotes manganari* sp. n. (Ciliophora, Euplotida) isolated from the Sea of Marmara, Istanbul (Turkey). Protistology.

[21] Do E.-H., Kwon H.-I., Yeo J.H., Quintela-Alonso P., Jung J.-H. (2024). Morphology and molecular phylogeny of *Euplotes baugilensis* n. sp. (Ciliophora, Spirotrichea), with an illustrated key to *Euplotes* species with reduced cirri. Eur. J. Protistol..

[22] Abraham J.S., Somasundaram S., Maurya S., Gupta R., Makhija S., Toteja R. (2021). Characterization of *Euplotes lynni* nov. spec., *E. indica* nov. spec. and description of *E. aediculatus* and *E. woodruffi* (Ciliophora, Euplotidae) using an integrative approach. Eur. J. Protistol..

[23] Wang Y., Yan Y., Yi Z., Zhao Y., Zhang Q., Chi Y., Zhang T., Liu W., Liu M., Lu B. (2026). Comprehensive phylogenomic analyses of ciliated protists with a revised classification of the phylum Ciliophora (Eukaryota, Alveolata). Sci. China Life Sci..

[24] Chi Y., Wei F., Tang D., Mu C., Ma H., Wang Z., Al-Rasheid K.A.S., Hines H.N., Chen X. (2024). Exploring the biogeography, morphology, and phylogeny of the condylostomatid ciliates (Alveolata, Ciliophora, Heterotrichea), with establishment of four new *Condylostoma* species and a revision including redescriptions of five species found in China. Mar. Life Sci. Technol..

[25] Obert T., Zhang T., Rurik I., Vďačný P. (2025). Rediscovery and morpho-molecular characterization of three astome ciliates, with new insights into eco-evolutionary associations of astomes with their annelid hosts. Mar. Life Sci. Technol..

[26] Ma M., Tang D., Song W., Li L., Dovgal I.V., Al-Rasheid K.A.S., Hines H.N., Yan Y. (2024). Investigating the psammophilic karyorelictean ciliate families Kentrophoridae and Cryptopharyngidae (Protista, Ciliophora): Molecular phylogeny, geographic distributions and a brief revision including descriptions of a new genus, a new species and a new combination. Mar. Life Sci. Technol..

[27] Liu M., Jiang L., Zhang Z., Wei F., Ma H., Chen Z., Al-Rasheid K.A.S., Hines H.N., Wang C. (2024). Linking multi-gene and morphological data in the subclass Scuticociliatia (Protista, Ciliophora) with establishment of the new family Homalogastridae fam. nov. Mar. Life Sci. Technol..

[28] Dobri N., Ngueng Oumarou E., Alimenti C., Vallesi A. (2014). Polar and non-polar species of the protozoan ciliate *Euplotes* behave differently in response to environmental oxidative stress. Ital. J. Zool..

[29] Valbonesi A., Luporini P. (1990). Description of two new species of *Euplotes* and *Euplotes rariseta* from Antarctica. Polar Biol..

[30] Wilbert N., Song W. (2008). A further study on littoral ciliates (Protozoa, Ciliophora) near King George Island, Antarctica, with description of a new genus and seven new species. J. Nat. Hist..

[31] Petz W., Song W., Wilbert N. (1995). Taxonomy and ecology of the ciliate fauna (protozoa, Ciliophora) in the endopagial and pelagial of the Weddell Sea, Antarctica. Stapfia.

[32] Wilbert N. (1975). Eine verbesserte Technik der Protargolimpragnation fur Ciliaten. Mikrokosmos.

[33] Pan X., Bourland W.A., Song W. (2013). Protargol synthesis: An in-house protocol. J. Eukaryot. Microbiol..

[34] Foissner W. (2014). An update of ‘basic light and scanning electron microscopic methods for taxonomic studies of ciliated protozoa’. Int. J. Syst. Evol. Microbiol..

[35] Rosati G., Modeo L., Melai M., Petroni G., Verni F. (2004). A multidisciplinary approach to describe protists: A morphological, ultrastructural, and molecular study on *Peritromus kahli* Villeneuve-Brachon, 1940 (Ciliophora, Heterotrichea). J. Eukaryot. Microbiol..

[36] Medlin L., Elwood H.J., Stickel S., Sogin M.L. (1988). The characterization of enzymatically amplified eukaryotic 16S-like rRNA-coding regions. Gene.

[37] Hall T.A. (1999). BioEdit: A user-friendly biological sequence alignment editor and analysis program for Windows 95/98/NT. Nucleic Acids Symp. Ser..

[38] Stamatakis A. (2014). RAxML version 8: A tool for phylogenetic analysis and post-analysis of large phylogenies. Bioinformatics.

[39] Miller M.A., Pfeiffer W., Schwartz T. The CIPRES science gateway: Enabling high-impact science for phylogenetics researchers with limited resources. Proceedings of the 1st Conference of the Extreme Science and Engineering Discovery Environment: Bridging from the Extreme to the Campus and Beyond.

[40] Ronquist F., Teslenko M., van der Mark P., Ayres D.L., Darling A., Höhna S., Larget B., Liu L., Suchard M.A., Huelsenbeck J.P. (2012). MrBayes 3.2: Efficient Bayesian phylogenetic inference and model choice across a large model space. Syst. Biol..

[41] Nylander J.A.A. (2004). MrModeltest v2. Program Distributed by the Author.

[42] Kumar S., Stecher G., Li M., Knyaz C., Tamura K. (2018). MEGA X: Molecular evolutionary genetics analysis across computing platforms. Mol. Biol. Evol..

[43] Rajter L., Vd’ačný P. (2016). Rapid radiation, gradual extinction and parallel evolution challenge generic classification of spathidiid ciliates (Protista, Ciliophora). Zool. Scr..

[44] Hillis D.M., Bull J.J. (1993). An empirical test of bootstrapping as a method for assessing confidence in phylogenetic analysis. Syst. Biol..

[45] Alfaro M.E., Zoller S., Lutzoni F. (2003). Bayes or bootstrap? A simulation study comparing the performance of Bayesian Markov chain Monte Carlo sampling and bootstrapping in assessing phylogenetic confidence. Mol. Biol. Evol..

[46] Dragesco J. (1970). Cilies libres du Cameroun. Ann. Fac. Univ. Cameroun.

[47] Dragesco J. (1972). Ciliés libres de l’Ouganda. Ann. Fac. Sci. Univ. Féd. Cameroun.

[48] Dragesco J., Dragesco-Kernéis A. (1986). Ciliés libres de L’Afrique Intertropicale. Introduction à la Connaissance et à L’étude des Ciliés.

[49] Dragesco J. (2003). Infaciliature et morphometrie de vingt espèces de ciliés hypotriches recoltès au Rwanda et Burundi, comprenant *Kahliella quadrinucleata* n. sp., *Pleurotricha multinucleata* n. sp. et *Laurentiella bergeri* n. sp.. Trav. Mus. Nat. Hist. Nat..

[50] Liu M., Fan Y., Miao M., Hu X., Al-Rasheid K.A.S., Al-Farraj S.A., Ma H. (2015). Morphological and morphogenetic redescriptions and SSU rRNA gene-based phylogeny of the poorly-known species *Euplotes amieti* Dragesco, 1970 (Ciliophora, Euplotida). Acta Protozool..

[51] Wrześniowski A. (1870). Beobachtungen über Infusorien aus der Umgebung von Warschau. Z. Wiss. Zool..

[52] Ma H., Gong J., Wang Y., Hu X., Ma H., Song W. (2000). Morphological studies on *Euplotes eurystomus* (Ciliophora, Hypotrichida) compared with its related species from freshwater biotopes. J. Zibo. Univ..

[53] Lokhande S., Nikam S., Sontakke T., Bandar V., Bansode V. Morphological studies on soil protozoa *Euplotes eurystomus* from Godavari basin area at Paithan District. Proceedings of the National Conference on Advances in Bioscience & Environmental Science: Present & Future (ABES).

[54] Wise B.N. (1965). The morphogenetic cycle in *Euplotes eurystomus* and its bearing on problems of ciliate morphogenesis*. J. Protozool..

[55] Ruffolo J.J. (1976). Cortical morphogenesis during the cell division cycle in *Euplotes*: An integrated study using light optical, scanning electron and transnlission electron microscopy. J. Morphol..

[56] Fokin S.I., Di Giuseppe G., Erra F., Dini F. (2008). *Euplotespora binucleata* n. gen., n. sp. (Protozoa: Microsporidia), a parasite infecting the hypotrichous ciliate *Euplotes woodruffi*, with observations on microsporidian infections in Ciliophora. J. Eukaryot. Microbiol..

[57] Tuffrau M. (1960). Révision du genre *Euplotes*, fondée sur la comparaison des structures superficielles. Hydrobiologia.

[58] Carter H.P. (1972). Infraciliature of eleven species of the genus *Euplotes*. Trans. Amer. Micros. Soc..

[59] Hill B.F., Reilly J.A. (1976). A comparative study of three fresh-water *Euplotes* species (Ciliophora, Hypotrichida). Trans. Am. Microsc. Soc..

[60] Foissner W. (1982). Ökologie and Taxonomie der Hypotrichida (Protozoa: Ciliophora) einiger österreichischer Böden. Arch. Protistenkd..

[61] Dai R., Xu K., He Y. (2013). Morphological, physiological, and molecular evidences suggest that *Euplotes parawoodruffi* is a junior synonym of *Euplotes woodruffi* (Ciliophora, Euplotida). J. Eukaryot. Microbiol..

[62] Pierson B.F. (1943). A comparative morphological study of several species of *Euplotes* closely related to *Euplotes patella*. J. Morphol..

[63] Hosoi M. (1973). Scanning electron microscopy of *Euplotes woodruffi* (Ciliata). Zool. Mag. Tokyo.

[64] Shi X., Wang H. (1989). Study on morphology in *Euplotes woodruffi* by using modified silver techniques. Acta Zool. Sin..

[65] Song W., Bradbury P.C. (1997). Comparative studies on a new brachish water *Euplotes*, *E. parawoodruffi* n. sp., and a redescription of *Euplotes woodruffi* Gaw, 1939 (Ciliophora; Hypotrichida). Arch. Protistenkd..

[66] Shen Z., Lin X., Li J. (2008). Morphological redescription of a rare marine Euplotids, *Euplotes parawoodruffi* (Protozoa, Ciliophora, euplotida). Acta Zootaxon. Sin..

[67] Pierson B.F., Gierke R., Fisher A.L. (1968). Clarification of the taxonomic identification of *Euplotes eurystomus* Kahl and *E. aediculatus* Pierson. Trans. Am. Microsc. Soc..

[68] Pang Y., Wei H. (1999). Studies on the morphology and morphogenesis in *Euplotes aediculatus*. J. East China Norm. Univ. (Nat. Sci.).

[69] Foissner W., Blatterer H., Berger H., Kohmann F. (1991). Taxonomische und ökologische Revision der Ciliaten des Saprobiensystems. Band 1: Cyrtophorida, Oligotrichida, Hypotrichia, Colpodea..

[70] Zhang X., Wang Y., Fan Y., Luo X., Hu X., Gao F. (2017). Morphology, ontogeny and molecular phylogeny of *Euplotes aediculatus* Pierson, 1943 (Ciliophora, Euplotida). Biodivers. Sci..

[71] Tirjakova E., Botlikova S., Vďačný P. (2015). Checklist and distribution of ciliates from the family Euplotidae Ehrenberg, 1838 (Protista: Ciliophora: Spirotrichea) in Slovakia, central Europe. Zootaxa.

[72] Agamaliev F.G. (1967). Faune des ciliés mésopsammiques de la côte ouest de la mer Caspienne. Cah. Biol. Mar..

[73] Kahl A., Friedrich D. (1932). Urtiere oder Protozoa I: Wimpertiere oder Ciliata (Infusoria) 3. Spirotricha.

[74] Borror A.C. (1968). Systematics of *Euplotes* (Ciliophora, Hypotrichida); toward union of the old and the new. J. Protozool..

[75] Dujardin F. (1841). Histoire Naturelle des Zoophytes: Infusoires.

[76] Curds C.R. (1974). Descriptions of three species of *Euplotes* (Protozoa: Ciliatea). Bull. Br. Mus. Nat. His. (Zool.).

[77] Jones E., Owen G. (1974). New species of protozoa from Mobile Bay, Alabama. J. Mar. Sci..

[78] Fotedar R., Stoeck T., Filker S., Fell J.W., Agatha S., Al Marri M., Jiang J. (2016). Description of the Halophile *Euplotes qatarensis* nov. spec. (Ciliophora, Spirotrichea, Euplotida) Isolated from the Hypersaline Khor Al-Adaid Lagoon in Qatar. J. Eukaryot. Microbiol..

[79] Syberg-Olsen M., Irwin N., Vannini C., Erra F., Di Giuseppe G., Boscaro V. (2016). Biogeography and character evolution of the ciliate genus *Euplotes* (Spirotrichea, Euplotia), with description of *Euplotes curdsi* sp. nov. PLoS ONE.

[80] Lian C., Liu M., Li H., Wang L., Pan X. (2026). Taxonomy and phylogeny of three euplotid ciliates isolated from northeast of China: *Euplotes heilongjiangensis* n. sp., *E. daqingensis* n. sp., and *E.* cf. *daidalos* (Alveolata, Ciliophora, Euplotida). BMC Microbiol..

[81] Vallesi A., Giuseppe G.D., Dini F., Luporini P. (2008). Pheromone evolution in the protozoan ciliate, *Euplotes*: The ability to synthesize diffusible forms is ancestral and secondarily lost. Mol. Phylogenet. Evol..

[82] Zhao Y., Yi Z., Warren A., Song W. (2018). Species delimitation for the molecular taxonomy and ecology of the widely distributed microbial eukaryote genus *Euplotes* (Alveolata, Ciliophora). Proc. R. Soc. B Biol. Sci..

[83] Boscaro V., Syberg-Olsen M.J., Irwin N.A.T., Del Campo J., Keeling P.J. (2019). What can environmental sequences tell us about the distribution of low-rank taxa? The case of *Euplotes* (Ciliophora, Spirotrichea), including a description of *Euplotes enigma* sp. nov. J. Eukaryot. Microbiol..

